# Effect of the Milling Time of the Precursors on the Physical Properties of Sprayed Aluminum-Doped Zinc Oxide (ZnO:Al) Thin Films

**DOI:** 10.3390/ma5081404

**Published:** 2012-08-16

**Authors:** Thrinath Reddy Ramireddy, Velmurugan Venugopal, Jagadeesh Babu Bellam, Arturo Maldonado, Jaime Vega-Pérez, Subramaniam Velumani, María De La Luz Olvera

**Affiliations:** 1 Center for Nanotechnology Research, Vellore Institute of Technology, Vellore 632014, Tamil Nadu, India; E-Mails: thrinath87@gmail.com (T.R.R.); vvelmurugan@vit.ac.in (V.V.); 2 Department of Electrical Engineering-SEES, CINVESTAV-IPN, Apdo. Postal 14-740, México D.F. 07000, Mexico; E-Mails: omphy.laser@gmail.com (J.B.B.); amaldo@cinvestav.mx (A.M.); jvegape@hotmail.com (J.V.-P.); velu@cinvestav.mx (S.V.)

**Keywords:** zinc oxide, thin films, ultrasonic spray pyrolysis, transparent conductive oxides, TCO

## Abstract

Aluminum doped zinc oxide (ZnO:Al) thin films were deposited on soda-lime glass substrates by the chemical spray technique. The atomization of the solution was carried out by ultrasonic excitation. Six different starting solutions from both unmilled and milled Zn and Al precursors, dissolved in a mix of methanol and acetic acid, were prepared. The milling process was carried out using a planetary ball mill at a speed of 300 rpm, and different milling times, namely, 15, 25, 35, 45, and 60 min. Molar concentration, [Al]/[Zn] atomic ratio, deposition temperature and time, were kept at constant values; 0.2 M, 3 at.%, 475 °C, and 10 min, respectively. Results show that, under the same deposition conditions, electrical resistivities of ZnO:Al thin films deposited from milled precursors are lower than those obtained for films deposited from unmilled precursors. X-ray diffraction analysis revealed that all films display a polycrystalline structure, fitting well with the hexagonal wurtzite structure. Changes in surface morphology were observed by scanning electron microscopy (SEM) as well, since films deposited from unmilled precursors show triangular shaped grains, in contrast to films deposited from 15 and 35 min milled precursors that display thin slices with hexagonal shapes. The use of milled precursors to prepare starting solutions for depositing ZnO:Al thin films by ultrasonic pyrolysis influences their physical properties.

## 1. Introduction

ZnO thin films, deposited by chemical techniques, have recently received a great deal of attention due to their high-competitive quality with films processed by physical techniques [1,2]. These films have been used for different optoelectronic applications, such as solar cells [3], gas sensors [4], transparent electrodes [5], bacterial inactivation and degradation of organic wastes in water by photo-catalytic processes [6,7], together with others.

Spray pyrolysis is one most employed chemical deposition techniques, which has attracted great attention due to the fact that conductive and transparent ZnO thin films can be deposited in a simple and direct way, and no extra annealing step is required, as occurs in other chemical techniques [8]. In order to continually improve this technique, some changes have been made in the deposition systems, such as the use of an ultrasonic atomization that according to our experience leads to an enhancement in the transport properties of chemically sprayed thin films [9].

In meeting the challenge of obtaining highly conductive and transparent semiconductor thin films, a lot of studies have been conducted into the factors affecting the physical characteristics of the films. Based on these studies, it has been shown that, among the most important factors affecting the physical characteristics of the films are the starting solution conditions. In this respect, it has been reported that the use of different starting precursors affects in an important way the physical properties of the ZnO thin films, and particularly their surface morphology, a key aspect in the performance of conductive electrodes in thin film solar cells applications [10].

On the other hand, the development of materials in nano-particle size has been explored by using the ball milling technique [11]. Based on experimental results and supported by mathematical models, it has been stated that the ball milling process changes the nature of the starting reagents, since the milling induces a solid state reaction [12].

By using this simple milling process, and starting from zinc oxide and aluminum powders, nanoparticles of ZnO:Al have been synthesized by other researchers [13]. However, to our best knowledge, the effect of milling on the precursors in chemically sprayed ZnO:Al thin films has not been reported.

The present work reports the effect of the milling time of the starting precursors on the electrical, structural, morphological, and optical characteristics of ZnO:Al thin films deposited on soda lime glass substrates by the ultrasonic spray pyrolysis technique (USP). ZnO:Al thin films from unmilled precursors were also deposited for comparison and to investigate the milling effect on the physical characteristics of the films.

## 2. Experimental

Six starting solutions from unmilled and milled powders of a mix of hydrated zinc acetate [Zn(CH_3_COO)_2_·2H_2_O, Alfa-Aesar, Ward Hill, MA, USA] and aluminum pentanedionate [Al(CH_3_COCHCOCH_3_)_3_, Merck, Whitehouse Station, NJ, USA] for depositing ZnO:Al thin films were prepared. Constant values of molar concentration (0.2 M) and doping atomic ratio ([Al/Zn] = 3 at.%), were used. The milling process was carried out by a Retch PM400 planetary ball milling equipment at a constant velocity of 300 rpm with different times, namely, 15, 25, 35, 45 and 60 min. ZnO:Al thin films from unmilled precursors were deposited as reference films. The milled and unmilled precursors were dissolved separately in a mix of methanol and acetic acid (90:10 volume proportion). A stirring process was used to obtain complete dissolution of the precursors. The addition of acetic acid [CH_3_CO_2_H, Baker, Xalostoc, Edo. De Mex., Mexico] was needed to avoid the early precipitation of zinc hydroxides. ZnO:Al thin films were prepared by the USP technique. The deposition system includes a piezoelectric transducer operating at a frequency of 1.2 MHz [Ultrasonic Humidifier HUM 006, Sunshine Co., México, Mexico] [14].

All films were deposited on 2.5 cm × 2.5 cm clean soda-lime glass substrates. The cleaning process was as follows: (i) five minutes in an ultrasonic bath in trichloroethylene to degrease the substrates; followed by (ii) five minutes in a bath in methyl alcohol; (iii) five minutes in an ultrasonic bath in acetone [CH_3_COCH_3_]; and finally (iv) a drying process with a gas nitrogen jet [N_2_]. The substrates were then placed on a melted tin bath, whose temperature is measured just below the substrate by using a thin chromel-alumel thermocouple contained in a stainless steel metal jacket. The substrate temperature was fixed at 475 °C, with an accuracy of ±1 °C. Gas nitrogen [N_2_] was used as carrier gas. The solution and carrier flow rates were held constant at values of 1 mL min^−1^, and 2 L min^−1^, respectively. A constant deposition time of 10 min led to films with a thickness around 600 nm. The film thicknesses were measured by a KLA Tencor P15 profilometer, after preparing a step by chemical etching in dilute hydrochloric acid.

Electrical characterization consisted of Hall Effect measurements by using the van Der Paw method in a magnetic field of 5000 G and an electrical current of a 10^−3^ A. The structure of the films was characterized by means of X-ray diffraction in an X´Pert Pro PANalytical system, by using the θ–2θ technique, based on the Cu-K_α_ radiation (λ = 1.5405 Å). Surface morphology was analyzed with a JEOL JSM-35C scanning electron microscope (SEM). The optical transmittance at normal incidence was measured with a double-beam UV-Vis Shimadzu spectrophotometer, in the UV-visible region (300–1000 nm) without glass substrate correction.

## 3. Results and Discussion

### 3.1. Electrical Characteristics

**Figure 1:**
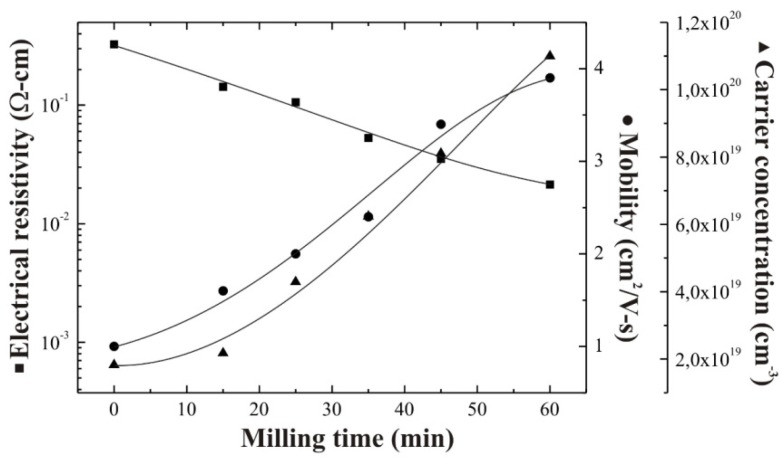
Electrical resistivity, electron mobility and carrier concentration as a function of the milling time of chemically sprayed ZnO:Al thin films.

Figure 1 shows the variation in the electrical resistivity, electron mobility, and carrier concentration of ZnO:Al thin films deposited from both unmilled and milled precursors at a constant substrate temperature, 475 °C. Samples deposited from unmilled precursors show a higher resistivity compared with those samples deposited from milled precursors, under the same deposition conditions. Additionally, as the milling time is increased a gradual resistivity decrease is clearly evidenced from Figure 1, reaching a maximum decrease around one order of magnitude after a milling time of 60 min. This trend can be associated with two different phenomena, firstly, a stoichiometric deviation present in the ZnO lattice, and secondly an increase in the Al incorporation efficiency into the ZnO lattice. In this respect, it is well-known that the n-type character of the ZnO, in massive or thin film form, is attributed to the stoichiometric deviation due to the simultaneous presence of oxygen vacancies and interstitial Zn atoms. It is worth mentioning that in this work the deposition temperature was kept constant at a value of 475 °C, however according to our experience we know that, temperature variation could lead to an additional resistivity decrease. In this respect, our future challenge is to reduce the film resistivity by optimizing the deposition temperature. Electron mobility values measured from the Hall effect are low, varying between 1 and 4 cm^2^/(V-s), which are typical values obtained in chemically sprayed ZnO films, because of the high density of defects present in films deposited by chemical techniques. According to electron mobilities and carrier concentration values measured, it can be considered that the carrier concentration is the main reason for the decrease in the electrical resistivity.

### 3.2. Structural Characteristics

**Figure 2:**
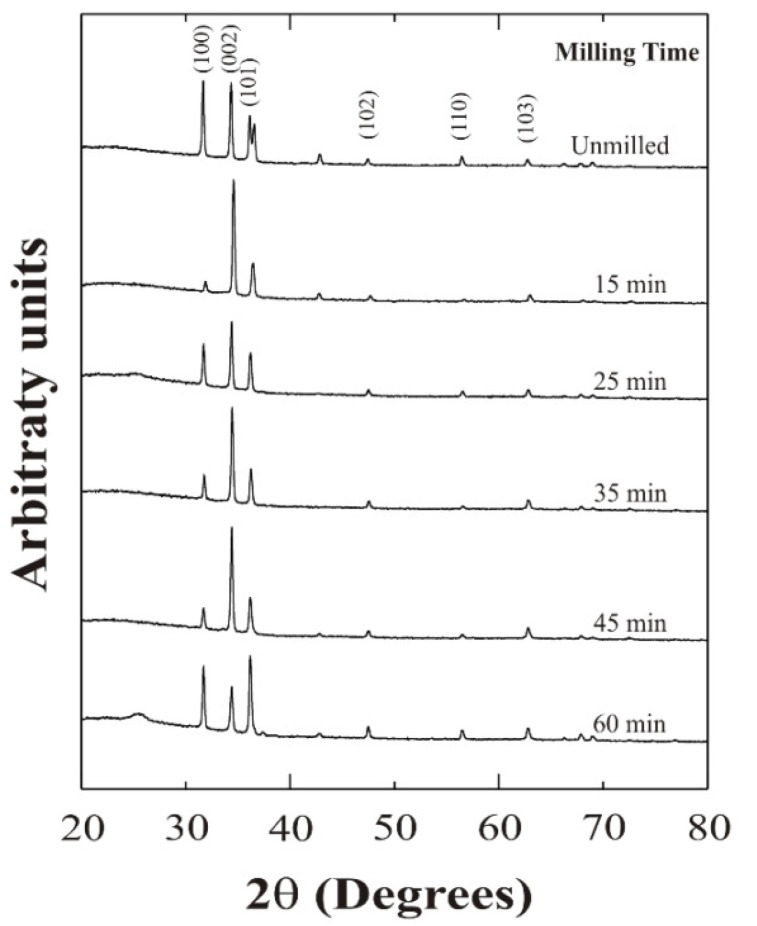
X-ray spectra of ZnO:Al thin films prepared from unmilled and milled Zn and Al precursors.

Figure 2 shows the X ray diffraction spectra of ZnO:Al thin films deposited from unmilled and milled precursors. The diffraction patterns reveal that all films are polycrystalline and fit well with the ZnO hexagonal wurtzite structure [15]. Samples deposited from 15 to 45 min milled precursors exhibited a stronger reflection along (002) plane, whereas the rest presented a random crystallinity with similar contribution of (100), (002), and (101) planes. It is interesting to note that depending on the deposition conditions an extra peak located at 42.4° not associated with ZnO, appears. According to the deposition conditions used, this peak could be associated with a zinc hydroxide compound present in the film, despite using an excess of acetic acid in the starting solutions to avoid this. In fact, in the case of samples deposited from 25 and 35 min milled precursors it does not appear; nevertheless its intensity is very small to be considered as an important phase. It is believed that, by the milling process of the precursors two effects are possible; firstly, removal of the water content from the zinc acetate, and secondly, stimulation of a solid state reaction of the mixed powders. Then, a variation of the milling time of the precursors results in films with different structural properties.

**Figure 3:**
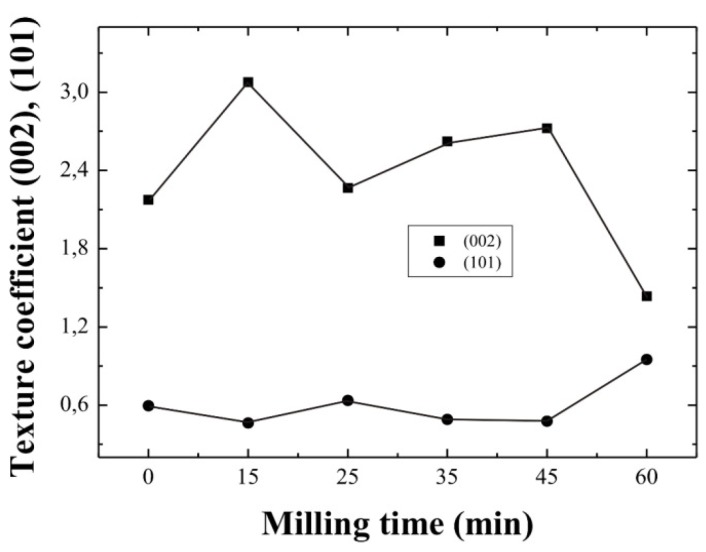
Dependence of the texture coefficient of ZnO:Al thin films on the milling time of the starting precursors.

In addition, changes in the intensity of the peaks associated with the crystalline planes are observed, depending on the milling time. In order to measure the preferential growth, the texture coefficient was calculated using the following mathematical expression [16]:

$$T_{c}(h\;k\;l)=\frac{\left[I(h\;k\;l)/I_{r}(h\;k\;l)\right]}{1/N\;\Sigma \;\left[I(h\;k\;l)/I_{r}(h\;k\;l)\right]}$$

where *T_c_(hkl)* is the corresponding texture coefficient, *I(hkl)* is the X-ray diffraction intensity obtained from the ZnO:Al thin films and *N* is the number of diffraction peaks. *I_r_(hkl)* is the intensity of the X-ray diffraction pattern reference (in this case from the JCPDS ZnO card). Figure 3 shows the variation of the *T_c_(hkl)* of the (002) and (101) planes as a function of the milling time for ZnO:Al thin films deposited from both unmilled and milled precursors. Thus, from texture coefficient calculations, the peak associated with the (002) planes of the ZnO films, prevails over the rest, as the *T_c_(hkl)* value is higher than one, except for the sample corresponding to a milling time of 60 min, where the (101) planes are dominant. Therefore, since these values are higher than one, it is possible to conclude that preferential growth in the corresponding (*hkl*) direction was obtained.

Additionally, crystallite sizes were determined based on (002) plane from XRD data of the samples. Here, the full width at half maximum (FWHM) was used in conjunction with the Debye-Scherer formula [17]:

$$D=\frac{0.9\;\lambda }{B\;\cos\theta }$$

where *D* is the crystallite size in nanometers; λ is the wavelength value of the Cu-K_α_ line (λ = 1.5406 Å); *θ* is the Bragg diffraction angle; and *B* is the FWHM of the diffraction peak measured in radians. The estimated crystallite size values oscillated in the range of 35 to 41 nm, and are reported in Table 1.

**Table 1:** Crystallite size.

Milling time (min)	Crystallite size (nm)
0 (unmilled)	41.1
15	35.6
25	36.4
35	36.6
45	35.0
60	35.1

### 3.3. Morphology

**Figure 4:**
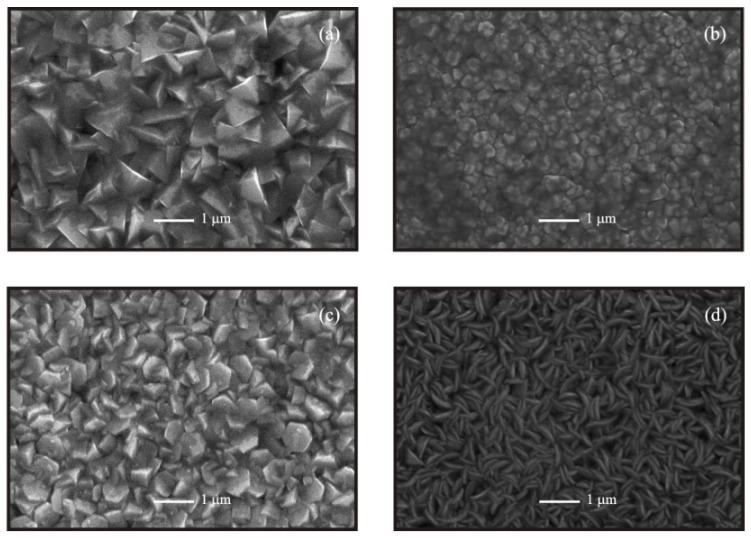
Scanning electron microscopy (SEM) images of ZnO:Al thin films deposited from starting solutions prepared from unmilled and milled precursors at different times: (**a**) unmilled; (**b**) 15 min; (**c**) 35 min; and (**d**) 60 min.

Figure 4 shows the surface morphology of ZnO:Al thin films deposited from unmilled, 15, 35 and 60 min milled precursors. It is worthy of note that grains with definite geometrical shape are formed in all samples. This characteristic needs to be highlighted since ultrasonic spray resembles chemical vapor deposition, where growth occurs during a steady state process. It is well-known that, to have control of the film morphology is extremely important in optoelectronic applications of ZnO thin films, with the particular advantage that, in the case of chemical spraying the cost of the set-up is low, compared with the CVD technique.

SEM micrographs show that, a dramatic change in morphology occurs due to the milling process of the precursors. As a matter of fact triangle-shaped or triangular pyramid-like grains are observed in the case of the ZnO:Al thin films deposited from unmilled precursors, while a change to poorly faceted hexagonal slices is observed in samples deposited from 15 min milled precursors. Films deposited from 35 min milled precursors show a surface covered by big and well-faceted hexagonal grains with a size around 750 nm. Finally, films deposited from 60 min milled precursors showed a surface morphology completely different from the rest in which a surface covered by features with a geometry of bulky last quarter moons is evidenced.

### 3.4. Optical Transmission

**Figure 5:**
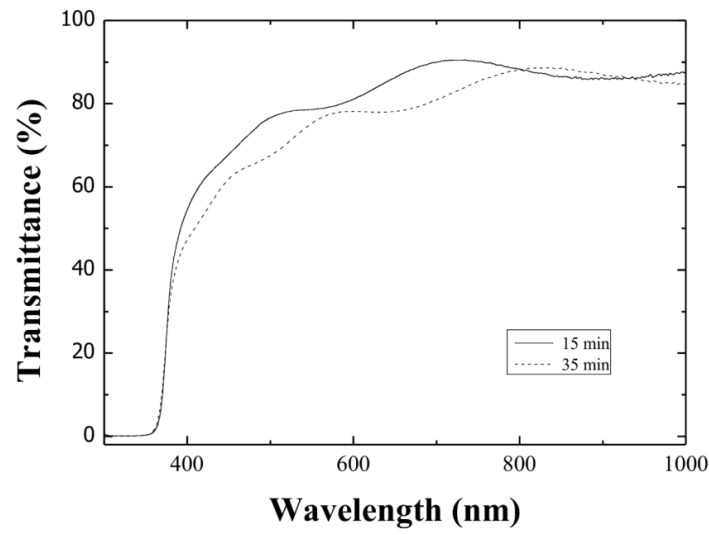
Optical transmission spectra of ZnO:Al thin films deposited from starting solutions prepared from 15 and 35 min milled precursors.

Figure 5 presents the optical transmission spectra of ZnO:Al thin films deposited from 15 and 35 min milled precursors. The average transmittance at 550 nm varied around 80% in all samples, which is an adequate value for transparent conductive electrodes. The optical band gap values, *E_G_*, were estimated from the absorption spectra using the well-known equation for direct band gap semiconductors, (*αhν*)^2^ = *C*(*hν* − *E_G_*), where *α* is the absorption coefficient, *hν* the photonic energy, and C is a constant. Hence, the *E_G_* values can be estimated by extrapolating the lineal portion for the energy axis in the corresponding (*hν*)^2^ versus hν graph, since for (*hν*)^2^ = 0, we have (*hν*) = *E_G_*. The band gap values were in the order of 3.3 eV, and no significant changes were observed with the milling time variation.

## 4. Conclusions

The role of the milling process of the precursors on the physical characteristics of ZnO:Al thin films deposited on sodocalcic glass substrates by an ultrasonic spray technique has been presented. The main registered effect was the decrease in electrical resistivity compared to those samples deposited from unmilled precursors, since a variation of around one order of magnitude was obtained. Thus, the milling of the precursors can be considered as a prior process that enhances the transport properties of chemically sprayed films. Additionally we can observe a change of the morphology with the milling time of the precursors. Another benefit of the milling of precursors is the complete formation of ZnO, with no extra phases of zinc compounds.

Therefore, the milling process offers interesting and technologically promising new possibilities, more than are currently utilized. However, more detailed work is necessary in order to determine the scope of the milling process. This work will be done in due course.
